# In-vivo assessment of myocardial calcium uptake using manganese-enhanced cardiovascular magnetic resonance in aortic stenosis

**DOI:** 10.1016/j.jocmr.2024.101074

**Published:** 2024-08-02

**Authors:** Abhishek Dattani, Saadia Aslam, Gaurav S. Gulsin, Aseel Alfuhied, Trisha Singh, Shruti S. Joshi, Lucy E. Kershaw, David E. Newby, Gerry P. McCann, Anvesha Singh

**Affiliations:** a Department of Cardiovascular Sciences, University of Leicester and the NIHR Leicester Biomedical Research Centre, Glenfield Hospital, Leicester, UK; b College of Applied Medical Sciences, King Saud Bin Abdulaziz University for Health Sciences, Riyadh, Saudi Arabia; c Centre for Cardiovascular Science, University of Edinburgh, Edinburgh, UK

**Keywords:** Aortic stenosis, Calcium, Manganese-enhanced MRI

## Abstract

**Background:**

Dysregulated myocardial calcium handling has been demonstrated in ischemic, non-ischemic and diabetic cardiomyopathy. Manganese-enhanced MRI (MEMRI) provides a unique method to quantify in-vivo myocardial calcium uptake but no studies have so far utilized MEMRI in patients with aortic stenosis (AS). We sought to: 1) determine whether myocardial calcium uptake is perturbed in people with severe AS, and 2) assess change in calcium uptake following aortic valve replacement (AVR).

**Methods:**

In this prospective, pilot, case-control study, adults with severe AS underwent MEMRI before and after AVR. A group of healthy controls were also recruited. The primary outcome was the rate of manganese uptake (Ki) as assessed by Patlak modeling to act as a surrogate of myocardial calcium uptake. Comparison of Ki between groups was adjusted for age, body mass index (BMI) and systolic blood pressure.

**Results:**

Twenty-eight controls and ten subjects with severe AS (age 72 [61-75] years, 8 male, 7 symptomatic, valve area 0.81 [0.74-1.0] cm^2^) were recruited, with seven returning for repeat scans post-AVR. AS patients had higher BMI and blood pressure, and a greater incidence of hyperlipidemia compared to controls. Baseline left ventricular (LV) volumes were similar between the groups, but the AS patients had higher indexed left ventricular mass. Global longitudinal strain and peak early diastolic strain rate were lower in the AS group. There was no significant difference in Ki between patients with severe AS and controls (7.09 [6.33-8.99] vs. 8.15 [7.54-8.78] mL/100g of tissue/min, P=0.815). Following AVR, there was regression in indexed LV mass (68 [51-79] to 49 [47-65] g/m^2^, P=0.018) and mass-volume ratio (0.94 [0.80-1.13] to 0.74 [0.71-0.82] g/mL, P=0.028) but no change in Ki was seen (7.35 [6.81-8.96] to 7.11 [6.16-8.01] mL/100 g of tissue/min, P=0.499).

**Conclusions:**

Despite clear features of adverse LV remodeling and systolic dysfunction, patients with severe AS demonstrated no alteration in calcium uptake at baseline compared to controls. Moreover, AVR led to reverse LV remodeling but no notable change in calcium uptake was seen. This may suggest that altered myocardial calcium handling does not play a significant pathophysiological role in AS.

## Graphical abstract

**Figure:**
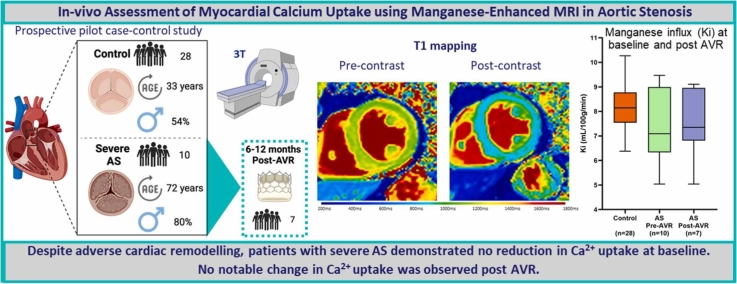
Created using BioRender.com

## 1 Introduction

Severe aortic stenosis (AS) causes cardiac remodeling leading to diastolic dysfunction, myocardial fibrosis, and microvascular dysfunction, with a poor prognosis without intervention once symptoms develop [1]. Traditionally, the focus of evaluation has been limited to the aortic valve, but it is recognized that the myocardial response to pressure overload is equally important in disease progression.

In pre-clinical models, dysregulated myocardial calcium (Ca^2+^) handling has been implicated in the development of heart failure in AS [2] but has not been studied in-vivo. Manganese is a paramagnetic Ca^2+^ analog for voltage-gated L-type Ca^2+^ channels found in cardiac myocytes. Manganese-enhanced cardiac magnetic resonance imaging (MEMRI) provides a novel method to quantify in-vivo myocardial Ca^2+^ uptake and has demonstrated impaired uptake in patients with ischemic [3], non-ischemic [4], and diabetic cardiomyopathies [5]. We sought to determine whether myocardial Ca^2+^ uptake is reduced in people with severe AS and to assess change in Ca^2+^ uptake following aortic valve replacement (AVR).

## 2 Methods

This was a prospective pilot case-control study. Adults with severe AS awaiting AVR were studied at baseline and 6–12 months following AVR. Exclusion criteria were other severe valve disease, cardiomyopathy, diabetes, arrhythmia, cardiac device, glomerular filtration rate <30 mL/min/1.73 m^2^, or contraindications to MEMRI. Healthy volunteers were also enrolled to act as a control group. The study was approved by the United Kingdom National Research Ethics Service (19/EM/0035, 17/WM/0192, 20/NS/0037, and 20/WM/0304) and participants provided written informed consent before study entry.

All participants underwent MEMRI scans performed using standardized protocols on 3T scanners (Skyra and Magnetom Skyrafit, Siemens Healthineers, Erlangen, Germany) with electrocardiographic gating and an 18-channel phased-array cardiac receiver coil. Cardiac structure and function were assessed as previously described [1]. In brief, after localizers, steady-state free precession cine images were acquired in four-, three-, and two-chamber views, and a stack of short-axis slices was obtained covering the entire left ventricle (LV). A pre-contrast T1 map in a mid-short-axis slice position was performed using a modified Look-Locker inversion recovery sequence (Siemens MyoMaps, Erlangen, Germany). An intravenous infusion of manganese dipyridoxyl diphosphate (5 µmol/kg at 1 mL/min; Exova SL Pharma, Wilmington, Delaware) was commenced and repeated T1 maps at the same location were performed every 2.5 min for 30 min.

Transthoracic echocardiography was performed on participants with AS by accredited sonographers to assess valve severity and diastolic function. Diastolic function was graded as per the American Society of Echocardiography guidelines [6].

Cardiovascular magnetic resonance imaging (CMR) image analysis was performed using cvi42 software (v5.10.1, Circle CVI, Calgary, Alberta, Canada) with cardiac chamber volumes, mass, and function quantified as previously described [1]. Tissue tracking was used to assess myocardial strain, as previously described [7], to calculate global longitudinal strain and longitudinal peak early diastolic strain rate. For analysis of manganese uptake, regions of interest were drawn in the LV blood pool and the inferoseptal segment for all T1 maps from 0 to 30 min, with avoidance of areas of focal fibrosis.

The primary outcome measure was myocardial manganese influx constant (Ki) calculated using Patlak modeling. A two-compartment model was used comprising a reversible compartment (arterial) and an irreversible compartment (myocardial tissue) with manganese concentrations derived from blood pool and myocardial T1 values, respectively [4].

### 2.1 Statistical analysis

Statistical analysis was performed using SPSS (Statistical Package for Social Sciences, v28.0, Chicago, Illinois,). Power calculations were performed using G*Power (v3.1.9.7, Heinrich-Heine-Universität Düsseldorf, Düsseldorf, Germany). To detect a 20% difference in Ki between the AS and control group, with a two-sided alpha level of 0.05 and a 1:2 ratio of participants, 9 patients with AS and 19 controls would be required to achieve 90% power.

Normality was assessed using histograms and the Shapiro-Wilk test. Continuous data were expressed as mean ± standard deviation if normally distributed, or median [25%–75% interquartile range] if not. Unadjusted values are presented but statistical comparison of imaging parameters between AS patients and controls were adjusted for age, body mass index, and systolic blood pressure using analysis of covariance. Pre- and post-AVR comparison was performed using the Wilcoxon signed-rank test.

## 3 Results

Ten patients with AS (age 72 [61–75] years, 8 male, 7 symptomatic, valve area 0.81 [0.74–1.0] cm^2^) and 28 control subjects (age 33 [26–54] years, 15 male) were recruited. Of the 10 AS patients, 3 did not return for post-AVR assessments as they declined (n = 1), had a pacemaker implanted (n = 1), or contrast was not available (n = 1).

The AS group had higher body mass index (29 [27–31] versus 25 [24–28] kg/m^2^) and systolic blood pressure (166 [146–171] versus 125 [122–132] mmHg) and greater incidence of hyperlipidemia (70% (7/10) versus 4% (1/28)) compared to controls. Echocardiography in the AS group showed median E/A ratio 0.67 [0.57–1.00] and average E/e′ ratio 12.2 [8.8–13.6]. Three patients with AS met criteria for grade II/III diastolic dysfunction.

The AS group had similar cardiac volumes but greater indexed LV mass (68 [56–80] versus 54 [49–60] g/m^2^, P = 0.006) and a trend toward greater mass-to-volume ratio (0.89 [0.76–1.14] versus 0.70 [0.67–0.80] g/mL, P = 0.056) compared to control subjects (Table 1). Patients with AS also demonstrated impaired systolic and diastolic function: global longitudinal strain (15.6 [13.2–17.2] versus 18.5 [16.7–19.9] %, P < 0.001) and peak early diastolic strain rate (0.57 [0.45–0.86] versus 0.89 [0.82–1.04] s^−1^, P = 0.031).

**Table 1:** Comparison of baseline CMR parameters between the AS and control group.

	AS	Control	P value*
LV EDVi (mL/m^2^)	72 [63–103]	75 [68–83]	0.144
LV ESVi (mL/m^2^)	25 [18–36]	27 [22–31]	0.158
LV EF (%)	67.9 [60.7−69.6]	64.0 [60.9−68.0]	0.471
LVMi (g/m^2^)	68.2 [55.9−79.6]	53.8 [49.1−60.4]	**0.006**
LVM/EDV (g/mL)	0.89 [0.76−1.14]	0.70 [0.67−0.80]	0.056
GLS (%)	15.6 [13.2−17.2]	18.5 [16.7−19.9]	**<0.001**
PEDSR (s^−1^)	0.57 [0.45−0.86]	0.89 [0.82−1.04]	**0.031**

*CMR* cardiovascular magnetic resonance imaging, *AS* aortic stenosis, *EF* ejection fraction, *EDVi* indexed end-diastolic volume, *GLS* global longitudinal strain, *LV* left ventricle, *LVMi* indexed left ventricular mass, *PEDSR* longitudinal peak early diastolic strain rate, *ESVi* indexed end-systolic volume, *EDV* end-diastolic volume, *LVM* left ventricular mass.

Bold represents P value < 0.05.

* Adjusted for age, body mass index, and systolic blood pressure.

There was no difference in Ki between the AS group and control subjects after adjustment for age, body mass index, and systolic blood pressure (7.09 [6.33–8.99] versus 8.15 [7.54–8.78] mL/100 g of tissue/min, P = 0.815; Graphical Abstract). In a sensitivity analysis comparing baseline AS patients with 10 older control subjects (age 58 [53–61] years, 4 male), consistent findings were demonstrated with no difference in Ki between patients and controls (7.09 [6.33–8.99] versus 7.34 [6.94–8.88] mL/100 g of tissue/min, P = 0.562) adjusted for the same covariates.

Following AVR, there was regression in indexed LV mass (68 [51–79] to 49 [47–65] g/m^2^, P = 0.018) and mass-volume ratio (0.94 [0.80–1.13] to 0.74 [0.71–0.82] g/mL, P = 0.028) but no change in Ki was seen (7.35 [6.81–8.96] to 7.11 [6.16–8.01] mL/100 g of tissue/min, P = 0.499).

## 4 Conclusion

This is the first study to assess in-vivo myocardial Ca^2+^ handling in patients with AS using MEMRI. In this study, despite clear features of adverse LV remodeling and systolic dysfunction, patients with severe AS demonstrated no reduction in Ca^2+^ uptake at baseline. Moreover, AVR led to dramatic reverse LV remodeling but no notable change in Ca^2+^ uptake was seen. Dysregulated Ca^2+^ handling has been demonstrated in several types of cardiomyopathy 3, 4 and has recently been shown in asymptomatic patients with type 1 and type 2 diabetes [5] who are characterized by features of concentric remodeling and diastolic dysfunction.

Although these findings do not exclude alterations in Ca^2+^ handling in severe late-stage AS (only one participant met the criteria for very severe AS) [8], they suggest that Ca^2+^ handling does not play a significant role in the myocardial changes seen in AS which are primarily driven by pressure overload causing LV hypertrophy. This is contrary to the distinct changes seen in Ca^2+^ uptake in genetic and metabolic cardiomyopathies.

## 5 Strengths and Limitations

The strengths of this study are its novelty and prospective design with a pre-specified hypothesis. The AS patients all had severe AS and evidence of cardiac remodeling and dysfunction which is representative of the overall population with severe AS. Strict exclusion criteria, such as presence of other valve disease, cardiomyopathy, and diabetes, were also applied to both the AS and control groups.

There were some key limitations in this study. First, this was a pilot study and therefore the number of patients is relatively small, although it was reasonably powered to assess a difference between those with AS compared to controls. Second, AS participants were significantly older and had higher body mass index compared to controls, although we did account for this by statistical adjustments and a sensitivity analysis. We did not undertake gadolinium-enhanced MRI so were unable to assess the impact of focal or diffuse fibrosis, although areas of obvious fibrosis on T1 maps were avoided during contouring. We also acknowledge that there are some assumptions and limitations of Patlak modeling. For example, it is assumed that there is irreversible trapping of the manganese contrast within the intracellular compartment. The Patlak modeling calculations have been further optimized since our previous work which makes it difficult to make direct comparisons with published work in patients with hypertrophic and dilated cardiomyopathy [4] but is comparable to published literature in diabetes [5] and takotsubo cardiomyopathy [9].

## Funding

This study was funded by a 10.13039/501100000274British Heart Foundation (BHF) Leicester Accelerator Pump Priming Award. A.S. is funded by an 10.13039/100006662NIHR advanced fellowship (NIHR300867). The BHF supported A.D. (FS/CRTF/20/24069), S.S.J. (FS/CRTF/20/24087 and RE/18/5/34216), G.S.G. (FS/16/47/32190 and FS/TF/21/33008), and D.E.N. (CH/09/002, RG/20/10/34966, RE/18/5/34216, CH/F/21/90010). D.E.N. is the recipient of a 10.13039/100004440Wellcome Trust Senior Investigator Award (WT103782AIA). G.P.M. received funding from the NIHR through a Research Professorship award (RP-2017-08-ST2-007). The DAPA-MEMRI study was funded by an investigator-initiated award from 10.13039/100004325AstraZeneca (ESR-19-20118) and Pancreas MEMRI study was funded by the BHF (RE/18/5/34216). We acknowledge support from the NIHR Leicester Biomedical Research Centre and NIHR Leicester Clinical Research Facility. The Edinburgh Clinical Research Facilities and Edinburgh Imaging Facility are supported by the National Health Service Research Scotland through the National Health Service Lothian Health Board.

## Author contributions

**Abhishek Dattani:** Writing – original draft, Methodology, Investigation, Formal analysis. **Saadia Aslam:** Writing – review and editing, Methodology, Investigation, Formal analysis. **Gaurav S. Gulsin:** Writing – review and editing, Methodology, Investigation. **Aseel Alfuhied:** Methodology, Investigation. **Trisha Singh:** Methodology, Investigation. **Shruti S. Joshi:** Methodology, Investigation. **Lucy E. Kershaw:** Methodology, Investigation. **David E. Newby:** Writing – review and editing, Resources, Methodology, Investigation. **Gerry P. McCann:** Writing – review and editing, Supervision, Methodology, Conceptualization. **Anvesha Singh:** Writing – review and editing, Supervision, Funding acquisition, Conceptualization.

## Declaration of competing interests

The authors declare that they have no known competing financial interests or personal relationships that could have appeared to influence the work reported in this paper.
